# Supplementary material for: PSMA immunohistochemistry as a diagnostic biomarker of hepatocellular carcinoma
Source: JHEP Rep. 2025 Jul 31;7(11):101542. doi: 10.1016/j.jhepr.2025.101542 (PMC12529368; doi:10.1016/j.jhepr.2025.101542)
Supplement: Multimedia component 1 [file mmc1.pdf]

PSMA immunohistochemistry as a diagnostic biomarker of hepatocellular carcinoma

Killian Véron, Etienne Becht, Astrid Laurent-Bellue, Rémy Nicolle, Miguel Albuquerque, Samira Laouirem, Hélène Cazier, Clément Bailly, Mohamed Bouattour, Mickaël Lesurtel, Catherine Guettier, Rachida Lebtahi, Valérie Vilgrain, Valérie Paradis, Jérôme Cros, Aurélie Beaufrère

Table of contents

Table S1 2

Table S2 3

Table S3. 4

Table S4. 6

Table S5. 8

Fig. S1. 9

Fig. S2. 10

Fig. S3. 11

Table S1. List of morphological criteria assessed for each nodule and their definition.

Morphological criteria	Definition
Small-cell change	Cluster of small cells with decreased nucleo-cytoplasmic ratio
Large-cell change	Cluster of large cells with conserved nucleo-cytoplasmic ratio
Clone-like features (clear cells, fat)	Areas of clear cells and/or of cells containing fat
Increased cellular density	Nodule presented an increased cell density compared with extranodular cirrhotic liver
Presence of pseudoglands	Presence of pseudoglands or pseudorosettes architecture in the nodule
Loss of portal tracts	Absence of portal tracts in the nodule
Unpaired arteries	Presence of unpaired arteries in the nodule
Stromal invasion	Presence of tumour cells invading the fibrous tissue of portal tracts within or outside the nodule
Plate-thickening anomalies	Focal or diffuse increase of the thickness of the trabeculae (≥3 cells of thickness)
Alteration of the reticulin framework	Reduction or fragmentation or disappearance of the reticulinic framework

Table S2. Expression of PSMA, Glypican 3, HSP70, and GS across different nodule types and in the non-tumourous liver within the test cohort.

	PSMA+	Glypican 3+	HSP70+	GS+
Non-Cirrhotic Liver (n=79)	1 (1%)	2 (3%)	17 (22%)	1 (1%)
Cirrhosis (n=73)	4 (5%)	1 (1%)	6 (8%)	0 (0%)
RN (n=39)	7 (18%)	1 (3%)	2 (5%)	0 (0%)
LGDN (n=38)	8 (21%)	1 (3%)	2 (5%)	0 (0%)
HGDN (n=30)	8 (27%)	1 (3%)	3 (10%)	1 (3%)
eHCC (n=107)	82 (77%)	43 (40%)	45 (42%)	22 (21%)
HCC (n=106)	88 (83%)	69 (65%)	84 (79%)	31 (29%)

Table S3. Immunostaining performances of PSMA, Glypican 3, HSP70 and GS alone or in combination for diagnosing HCC within the test cohort (n=472).

Markers	Minimum of positive markers required	Se	Spe	PPV	NPV	Accuracy
PSMA	1	0.80	0.89	0.86	0.84	0.85
Glypican 3	1	0.53	0.98	0.95	0.71	0.77
HSP70	1	0.61	0.88	0.81	0.73	0.76
GS	1	0.25	0.99	0.96	0.62	0.66
PSMA, Glypican 3	1	0.86	0.87	0.84	0.88	0.81
PSMA, GS	1	0.84	0.88	0.86	0.87	0.86
PSMA, HSP70	1	0.89	0.78	0.77	0.90	0.83
Glypican 3, HSP70	1	0.73	0.86	0.82	0.80	0.81
Glypican 3, GS	1	0.62	0.97	0.94	0.76	0.81
HSP70, GS	1	0.68	0.88	0.82	0.77	0.79
PSMA, HSP70	2	0.51	1.00	1.00	0.71	0.78
PSMA, Glypican 3	2	0.46	1.00	1.00	0.69	0.71
Glypican 3, HSP70	2	0.40	1.00	0.99	0.67	0.73
PSMA, GS	2	0.21	1.00	1.00	0.61	0.64
HSP70, GS	2	0.18	1.00	0.97	0.60	0.63
Glypican 3, GS	2	0.15	1.00	1.00	0.59	0.62
PSMA, Glypican 3, GS	1	0.89	0.86	0.84	0.91	0.88
PSMA, HSP70, GS	1	0.90	0.77	0.76	0.90	0.83
PSMA, Glypican 3, HSP70	1	0.91	0.76	0.75	0.91	0.82

Glypican 3, HSP70, GS	1	0.78	0.86	0.82	0.83	0.82
PSMA, Glypican 3, HSP70	2	0.67	1.00	0.99	0.79	0.85
PSMA, HSP70, GS	2	0.61	1.00	0.99	0.75	0.82
PSMA, Glypican 3, GS	2	0.54	1.00	1.00	0.72	0.79
Glypican 3, HSP70, GS	2	0.47	0.99	0.98	0.69	0.76
PSMA, Glypican 3, HSP70	3	0.35	1.00	1.00	0.65	0.71
PSMA, Glypican 3, GS	3	0.15	1.00	1.00	0.59	0.61
PSMA, HSP70, GS	3	0.15	1.00	1.00	0.59	0.61
Glypican 3, HSP70, GS	3	0.13	1.00	1.00	0.58	0.61
PSMA, Glypican 3, HSP70, GS	1	0.92	0.75	0.75	0.92	0.83
PSMA, Glypican 3, HSP70, GS	2	0.73	0.99	0.99	0.82	0.88
PSMA, Glypican 3, HSP70, GS	3	0.41	1.00	1.00	0.67	0.73
PSMA, Glypican 3, HSP70, GS	4	0.12	1.00	1.00	0.58	0.60

Se, sensitivity, Sp, specificity, PPV, positive predictive value, NPV, negative predictive value

Table S4. Immunostaining performances of PSMA, Glypican 3, HSP70 and GS alone or in combination for diagnosing HCC in small nodules (≤2 cm) within the test cohort (n=213).

Markers	Minimum positive markers required	Se	Spe	PPV	NPV	Accuracy
PSMA	1	0.77	0.85	0.75	0.86	0.82
Glypican 3	1	0.40	0.98	0.91	0.73	0.76
HSP70	1	0.42	0.93	0.78	0.73	0.74
GS	1	0.21	0.99	0.96	0.68	0.70
PSMA, Glypican 3	1	0.80	0.83	0.74	0.88	0.82
PSMA, GS	1	0.79	0.84	0.75	0.87	0.82
PSMA, HSP70	1	0.80	0.78	0.68	0.87	0.79
Glypican 3, HSP70	1	0.56	0.91	0.79	0.78	0.78
Glypican 3, GS	1	0.48	0.97	0.91	0.76	0.79
HSP70, GS	1	0.51	0.93	0.81	0.76	0.77
PSMA, HSP70	2	0.38	1.00	1.00	0.73	0.77
PSMA, Glypican 3	2	0.36	1.00	1.00	0.73	0.76
Glypican 3, HSP70	2	0.26	0.99	0.97	0.69	0.72
PSMA, GS	2	0.19	1.00	1.00	0.67	0.70
Glypican 3, GS	2	0.13	1.00	1.00	0.66	0.68
HSP70, GS	2	0.11	0.99	0.92	0.65	0.67
PSMA, Glypican 3, GS	1	0.82	0.82	0.73	0.89	0.82
PSMA, HSP70, GS	1	0.82	0.78	0.69	0.88	0.79
PSMA, Glypican 3, HSP70	1	0.82	0.76	0.67	0.88	0.78

Glypican 3, HSP70, GS	1	0.63	0.91	0.81	0.80	0.80
PSMA, Glypican 3, HSP70	2	0.52	0.99	0.98	0.78	0.82
PSMA, HSP70, GS	2	0.46	0.99	0.98	0.76	0.79
PSMA, Glypican 3, GS	2	0.42	1.00	1.00	0.74	0.78
Glypican 3, HSP70, GS	2	0.30	0.99	0.94	0.70	0.73
PSMA, Glypican 3, HSP70	3	0.24	1.00	1.00	0.69	0.72
PSMA, Glypican 3, GS	3	0.13	1.00	1.00	0.66	0.68
PSMA, HSP70, GS	3	0.11	1.00	1.00	0.65	0.67
Glypican 3, HSP70, GS	3	0.10	1.00	1.00	0.65	0.67
PSMA, Glypican 3, HSP70, GS	1	0.84	0.76	0.68	0.89	0.79
PSMA, Glypican 3, HSP70, GS	2	0.57	0.99	0.97	0.79	0.83
PSMA, Glypican 3, HSP70, GS	3	0.28	1.00	1.00	0.70	0.73
PSMA, Glypican 3, HSP70, GS	4	0.10	1.00	1.00	0.65	0.67

Se: sensitivity, Sp: specificity, PPV: positive predictive value, NPV: negative predictive value

Table S5. Expression of PSMA, Glypican 3, HSP70, and GS across different nodule types and in the non-tumourous liver within the validation cohort.

	PSMA+	Glypican 3+	HSP70+	GS+
Non-Cirrhotic Liver (n=1)	0 (0%)	0 (0%)	1 (100%)	0 (0%)
Cirrhosis (n=36)	5 (14%)	0 (0%)	14 (39%)	0 (0%)
RN (n=22)	4 (18%)	0 (0%)	10 (45%)	0 (0%)
LGDN (n=16)	5 (31%)	1 (6%)	10 (63%)	0 (0%)
HGDN (n=8)	5 (63%)	0 (0%)	4 (50%)	0 (0%)
eHCC (n=24)	22 (92%)	9 (38%)	16 (67%)	8 (33%)
HCC (n=17)	17 (100%)	8 (47%)	15 (88%)	4 (24%)

Fig. S1. Examples of positive immunostainings in TMA HCC cases.

(A) Glutamine Synthetase: diffuse and intense expression of tumour cells, (B) Heat shock protein 70: nuclear expression of ≥5% of tumour cells, (C) Glypican 3: cytoplasmic and membranous expression of ≥5% of tumour cells, (D) Prostate Specific Membrane Antigen: expression of ≥5% of endothelial cells.

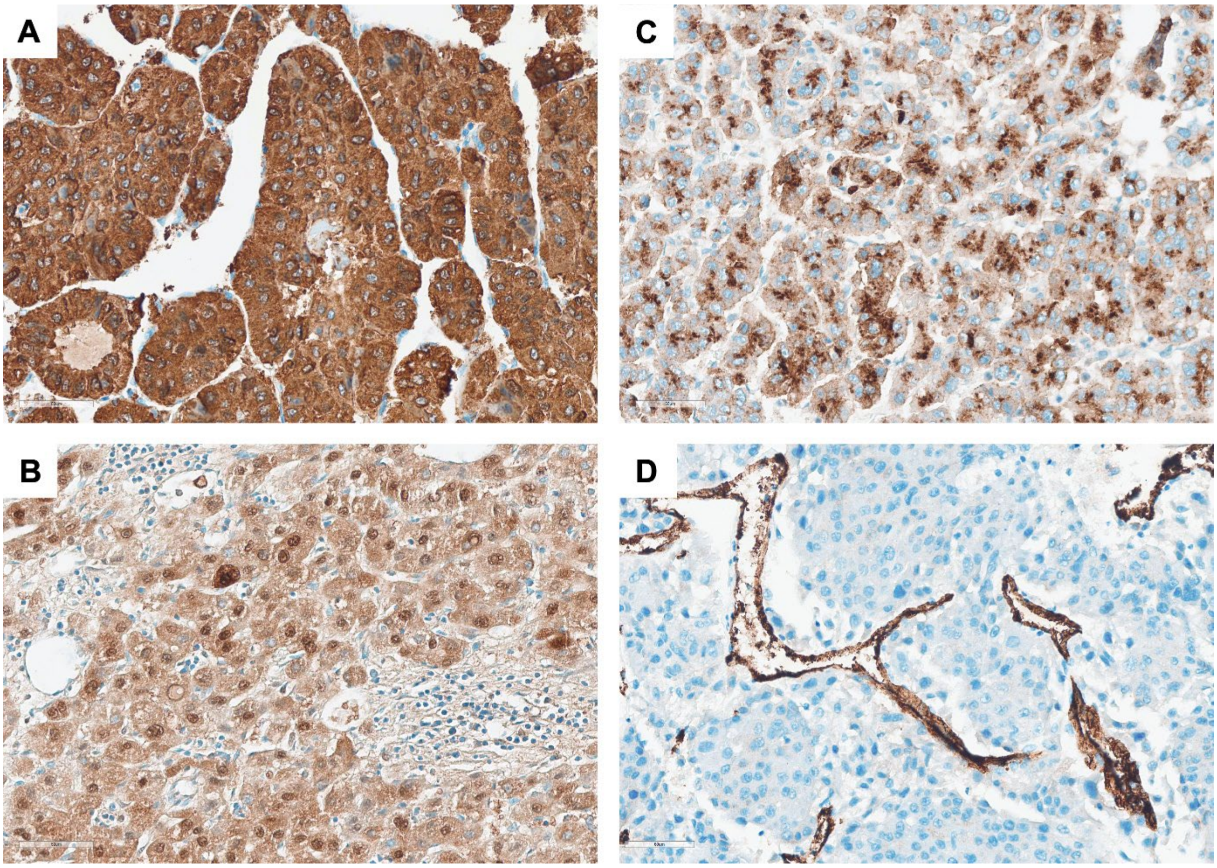

A case was considered positive if at least one core showed positive staining

Fig. S2. Venn Diagram of PSMA, Glypican 3, HSP70 and GS immunostainings in HCC cases from the test cohort (n=213).

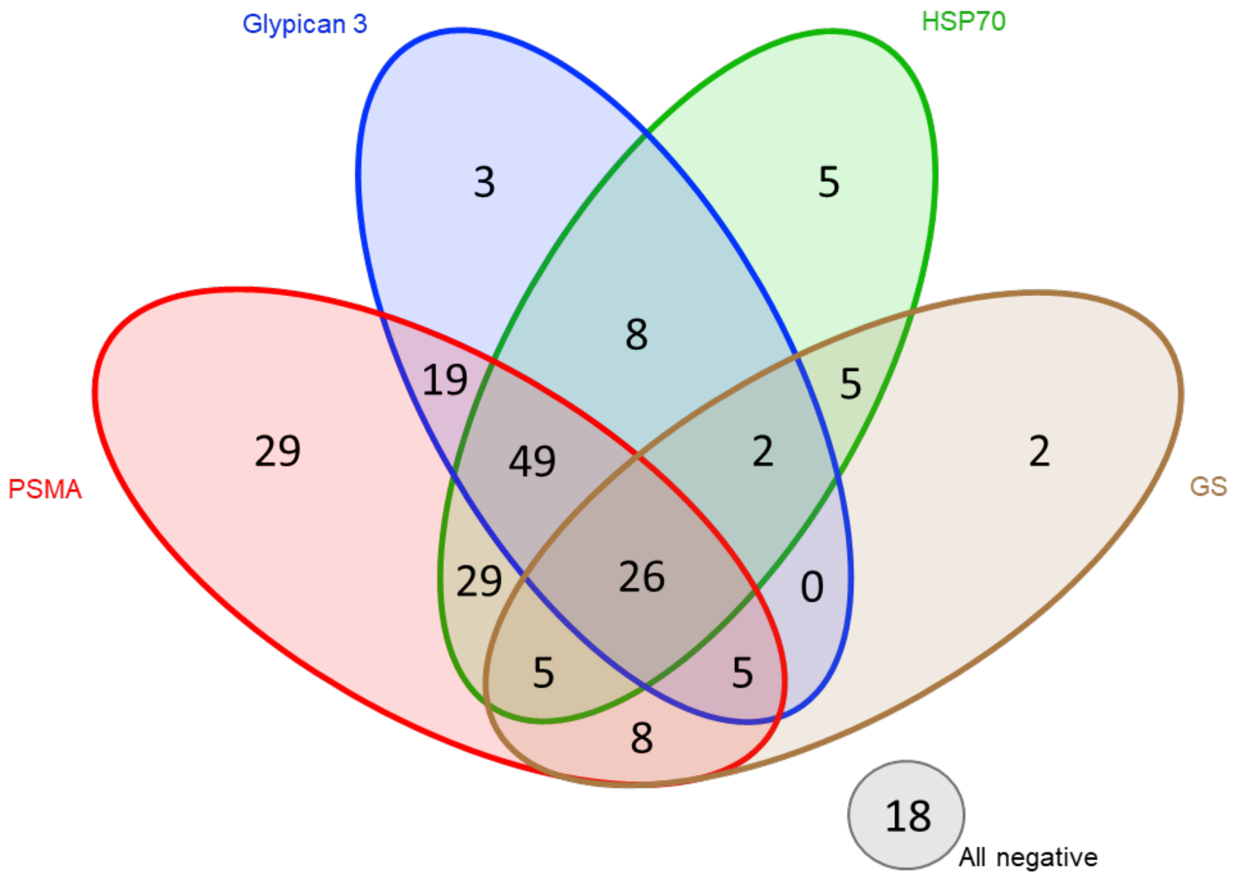

Fig. S3. PSMA expression in whole slides of paired surgical specimens (HCC and non-tumour liver) and biopsies from 15 cases.

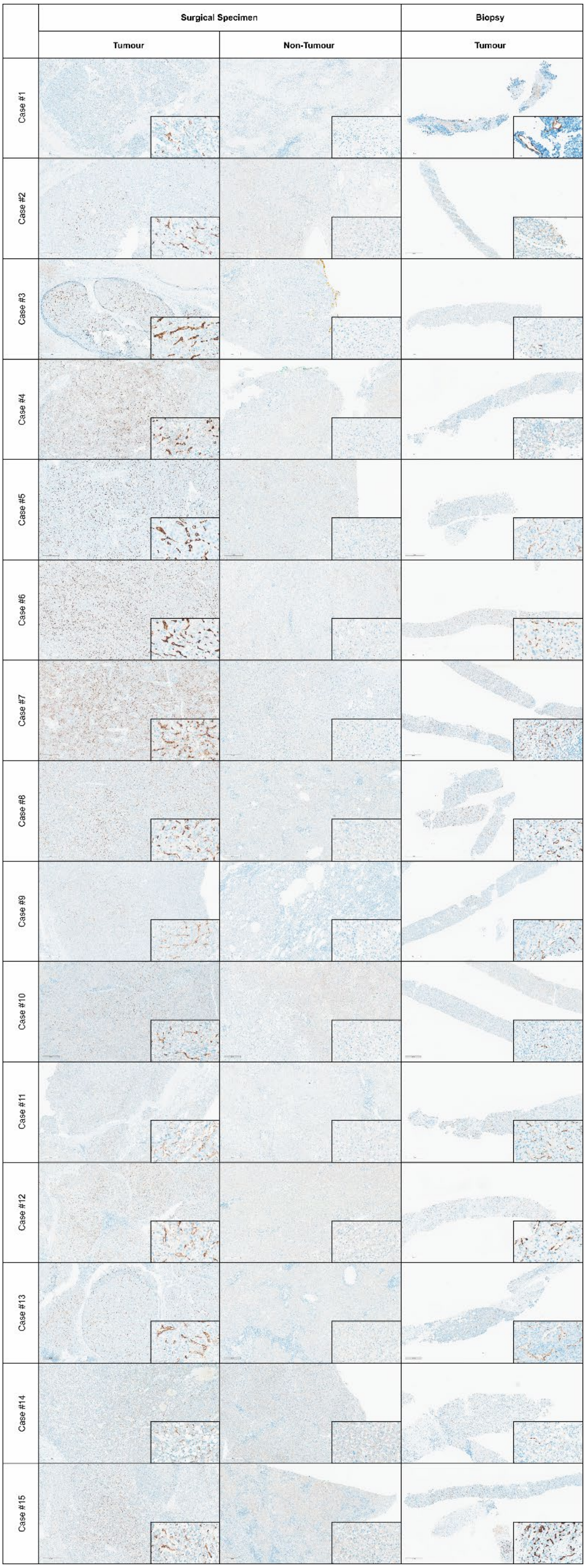
Surgical Specimen
Biopsy
Tumour
Non-Tumour
Tumour
Case #1
Case #2
Case #3
Case #4
Case #5
Case #6
Case #7
Case #8
Case #9
Case #10
Case #11
Case #12
Case #13
Case #14
Case #15
