# Supplementary material for: PSMA immunohistochemistry as a diagnostic biomarker of hepatocellular carcinoma
Source: JHEP Rep. 2025 Jul 31;7(11):101542. doi: 10.1016/j.jhepr.2025.101542 (PMC12529368; doi:10.1016/j.jhepr.2025.101542)
Supplement: Multimedia component 2 [file mmc2.docx]

**JHEP Reports**

**CTAT methods**

Tables for a “Complete, Transparent, Accurate and Timely account” (CTAT) are now mandatory for all revised submissions. The aim is to enhance the reproducibility of methods.

- Only include the parts relevant to your study
- Refer to the CTAT in the main text as ‘Supplementary CTAT Table’
- Do not add subheadings
- Add as many rows as needed to include all information
- Only include one item per row

**If the CTAT form is not relevant to your study, please outline the reasons why:**

|  |
| --- |

- 1. **Antibodies**

| **Name** | **Citation** | **Supplier** | **Cat no.** | **Clone no.** |
| --- | --- | --- | --- | --- |
| PSMA | PMID: 11092759 | Vitro Master Diagnóstica | MAD-000766QD-3 | EP192 |
| Glypican 3 | PMID: 17326147 | Zytomed | MSK067-5 | 1G12 |
| Hsp70 | PMID: 36829759 | Abcam | Ab5442 | 2A4 |
| Glutamine Synthetase | PMID: 7542742 | BD Biosciences | 610517 | 6/Glutamine Synthetase |

- 1. **Cell lines**

| **Name** | **Citation** | **Supplier** | **Cat no.** | **Passage no.** | **Authentication test method** |
| --- | --- | --- | --- | --- | --- |
|  |  |  |  |  |  |

- 1. **Organisms**

| **Name** | **Citation** | **Supplier** | **Strain** | **Sex** | **Age** | **Overall n number** |
| --- | --- | --- | --- | --- | --- | --- |
|  |  |  |  |  |  |  |

- 1. **Sequence based reagents**

| **Name** | **Sequence** | **Supplier** |
| --- | --- | --- |
|  |  |  |

- 1. **Biological samples**

| **Description** | **Source** | **Identifier** |
| --- | --- | --- |
|  |  |  |

- 1. **Deposited data**

| **Name of repository** | **Identifier** | **Link** |
| --- | --- | --- |
|  |  |  |

- 1. **Software**

| **Software name** | **Manufacturer** | **Version** |
| --- | --- | --- |
| Prism | GraphPad | 10.5 |
| Inkscape | Open-source | 1.4 |

- 1. **Other (*e.g*. drugs, proteins, vectors etc.)**

|  |  |  |
| --- | --- | --- |
|  |  |  |

- 1. **Please provide the details of the corresponding methods author for the manuscript:**

| Dr. Aurélie BEAUFRÈRE, aurelie.beaufrere@aphp.fr |
| --- |

**2.0 Please confirm for randomised controlled trials all versions of the clinical protocol are included in the submission. These will be published online as supplementary information.**

|  |
| --- |
