# Supplementary material for: PSMA immunohistochemistry as a diagnostic biomarker of hepatocellular carcinoma
Source: JHEP Rep. 2025 Jul 31;7(11):101542. doi: 10.1016/j.jhepr.2025.101542 (PMC12529368; doi:10.1016/j.jhepr.2025.101542)
Supplement: Multimedia component 4 [file mmc4.pdf]

Research article

JHEP Reports

PSMA immunohistochemistry as a diagnostic biomarker of hepatocellular carcinoma

Authors

Killian Véron, Etienne Becht, Astrid Laurent-Bellue, …, Valérie Paradis, Jérôme Cros, Aurélie Beaufrère

Correspondence

aurelie.beaufrere@aphp.fr (A. Beaufrère).

Graphical abstract

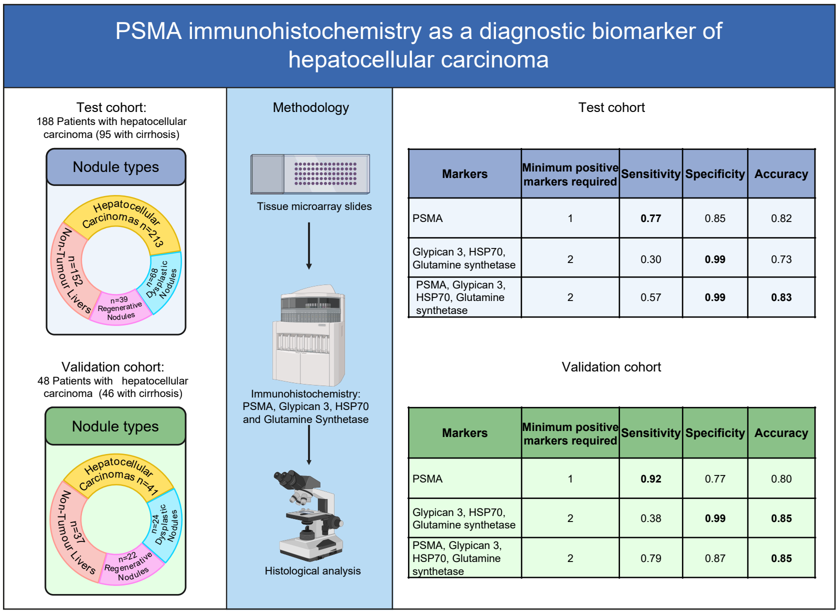

Highlights:

- PSMA demonstrates good diagnostic performance for HCC (sensitivity of 0.95 and specificity of 0.77).
- PSMA demonstrates superior diagnostic performance for HCC compared with routinely used markers.
- Adding PSMA to routine markers enhances sensitivity and accuracy.
- PSMA expression correlates with histoprognostic factors in HCC.

Impact and implications:

Differentiating hepatocellular nodules, particularly high-grade dysplastic nodules and hepatocellular carcinoma (HCC), based on histologic criteria remains challenging. In this study, we assess the diagnostic value of a new immunohistochemical marker, prostate-specific membrane antigen (PSMA), for diagnosing HCC in two independent series of hepatocellular nodules and compare its performance with that of routinely used markers (Glypican 3, heat shock protein 70 [HSP70], and glutamine synthetase [GS]). PSMA alone has demonstrated good performance in diagnosing HCC, superior to the combination of the three routinely used markers, and could be useful in practice for differentiating difficult-to-classify hepatocellular nodules. When the material is sparse, using PSMA alone could be recommended, whereas when sufficient material is available, adding PSMA to Glypican 3, HSP70, and GS may be advised, as this combination has shown the best performance for HCC diagnosis.

https://doi.org/10.1016/j.jhepr.2025.101542
© 2025 The Author(s). Published by Elsevier B.V. on behalf of European Association for the Study of the Liver (EASL). This is an open access article under the CC BY license (http://creativecommons.org/licenses/by/4.0/).

PSMA immunohistochemistry as a diagnostic biomarker of hepatocellular carcinoma

Killian Véron[1], Etienne Becht[1], Astrid Laurent-Bellue[2], Rémy Nicolle[1], Miguel Albuquerque[1,3], Samira Laouirem[1], Hélène Cazier[1], Clément Bailly[4], Mohamed Bouattour[1,5], Mickaël Lesurtel[1,6], Catherine Guettier[2], Rachida Lebtahi[1,7], Valérie Vilgrain[1,8], Valérie Paradis[1,3], Jérôme Cros[1,3], Aurélie Beaufrère[1,3,*]

JHEP Reports **2025**. vol. 7 | 1–10

Check for updates

Background & Aims: A combination of three immunohistological markers (Glypican 3, heat shock protein 70 [HSP70], and glutamine synthetase [GS]) is routinely used to differentiate hepatocellular carcinoma (HCC), but this panel's sensitivity is suboptimal. Our aim was to assess the diagnostic value of prostate-specific membrane antigen (PSMA) expression for diagnosing HCC in a series of hepatocellular nodules and compare its performance with that of routinely used markers.

Methods: We included 320 hepatocellular nodules from 188 patients in a test cohort and 87 hepatocellular nodules from 48 patients in an external validation cohort distributed as follows: regenerative nodules (RN, n = 39+22), low-grade dysplastic nodules (LGDN, n = 38+16), high-grade dysplastic nodules (HGDN, n = 30+8), early HCC (≤2-cm nodules) (n = 107+24), HCC (n = 106+17), and corresponding non-tumour livers (NTL, n = 152+37). PSMA, HSP70, Glypican 3, and GS expression was assessed by immunohistochemistry on tissue microarrays. For each marker or combination of markers, sensitivity, specificity, and accuracy were calculated.

Results: In the test cohort, PSMA was expressed in 83% of HCC (n = 88/106), 77% of early HCC (n = 82/107), 27% of HGDN (n = 8/30), 21% of LGDN (n = 8/38), 18% of RN (n = 7/39), and 3% of NTL (n = 5/152). In the validation cohort, the sensitivity and specificity of PSMA for HCC diagnosis were 0.95 and 0.77, respectively, and its accuracy was 0.83. The sensitivity and the specificity of the Glypican 3–HSP70–GS (≥2 positive markers) combination for HCC diagnosis were 0.41 and 0.99, respectively, and its accuracy was 0.80. Adding PSMA to this combination increased the sensitivity and accuracy to 0.85 and 0.86, respectively.

Conclusions: PSMA alone has shown good performance in diagnosing HCC, outperforming the combination of the three routinely used markers. When sufficient material is available, adding Glypican 3, HSP70, and GS to PSMA could be recommended.

© 2025 The Author(s). Published by Elsevier B.V. on behalf of European Association for the Study of the Liver (EASL). This is an open access article under the CC BY license (http://creativecommons.org/licenses/by/4.0/).

Introduction

Hepatocellular carcinoma (HCC), when developed on cirrhosis, is, most of the time, preceded by different steps of carcinogenesis, from large regenerative nodules (RN), to low-grade (LGDN) and high-grade (HGDN) dysplastic nodules.[1,2] Early diagnosis of HCC is critical, and differential diagnosis among these lesions, especially HGDN and early HCC, is challenging on imaging and biopsy. A dozen histological criteria are used to help classify nodules arising in cirrhotic liver, without assigning weights to each criterion and, in some cases, with poor inter-pathologist reproducibility.[1,3] In that context, additional staining, particularly an immunohistochemical panel of three antibodies (heat shock protein 70 [HSP70], glutamine synthetase [GS], and Glypican 3), is helpful, especially in an expert setting.[4,5]

HSP70 has a carcinogenic role owing to its antiapoptotic activity and was significantly overexpressed in early HCC tumour cells in comparison with hepatocytes in precancerous nodules.[6,7] Mutations on the third exon of the *CTNNB1* gene, which encodes β-catenin, are observed in around 20% of HCC and lead to nuclear translocation of β-catenin, which results in transcriptional upregulation of GS and its diffuse protein overexpression in tumour cells.[8,9] Glypican 3, a member of the glypican family of heparin sulfate proteoglycans, has been shown in numerous studies to be overexpressed in HCC tumour cells.[10–13] Di Tommaso *et al.*[14] demonstrated that the combination of these three biomarkers in the context of cirrhosis could be useful in diagnosing HCC. When at least two markers are positive, this combination demonstrates an excellent specificity (∼100%) but a moderate sensitivity

* Corresponding author. Address: Department of Pathology, Beaujon Hospital, 100 boulevard du Général Leclerc, Clichy, 92110, France. Tel.: +33-1-40-87-54-63; fax: +33-1-40-87-00-77.
E-mail address: aurelie.beaufrere@aphp.fr (A. Beaufrère).
https://doi.org/10.1016/j.jhepr.2025.101542

(47–72%) for the diagnosis of HCC.[4,5,14] The lowest sensitivity of this routine combination was observed in early HCC, which represents a significant limitation, as this type of nodule is the most challenging to diagnose.[15]

Prostate-specific membrane antigen (PSMA) is a transmembrane glycoprotein capable of transducing extracellular signals into the cytoplasm.[16] PSMA, originally found to be overexpressed in prostate cancer, has been considerably studied in recent decades for prostate cancer imaging and theragnostic applications.[17,18] Despite its name, PSMA expression is also observed in the neovasculature of a wide range of cancers other than prostate cancer, including digestive cancers such as oesophageal, gastric, pancreatic, and HCC.[19,20] PSMA plays a functional role in promoting angiogenesis within tumours, a process mediated by laminin substrates. In contrast, this function is absent in the endothelium of normal tissues.[19] Kmeid *et al.*[21] have recently highlighted the diagnostic interest of PSMA immunohistochemistry in hepatic neoplasms with excellent specificity and sensitivity for diagnosing HCC but with no comparison with markers used in routine and in a relatively restricted cohort of 164 cases, including only 68 HCC and 24 dysplastic nodules.

The aim of this study was to assess the diagnostic value of the immunohistochemical marker PSMA for diagnosing HCC and to compare its performance with that of routinely used markers in a large series of different types of hepatocellular nodules.

Materials and methods

Study population

We first selected 320 hepatocellular nodules from 188 patients with or without cirrhosis undergoing surgery between 2012 and 2021 in a French APHP reference centre (Beaujon Hospital, Clichy). Then, we constituted an external validation cohort from another French APHP reference centre (Bicêtre/Paul-Brousse Hospital), including 87 hepatocellular nodules from 48 patients. Fibrolamellar carcinomas and hepatocholangiocarcinomas were excluded from this study.

Written consent was obtained from all patients, as required by French legislation. The study was registered at the Commission Nationale de l'Informatique et des Libertés and approved by the local ethics committee (CER PARIS NORD no. 2020-015-IRB 00006477).

Data regarding demographics, risk factors for hepatocellular lesions, number of nodules, nodule size, and resection type were collected from electronic medical records and pathology reports.

Histologic review

Archived slides from nodules and non-tumour livers (including haematoxylin, eosin, and saffron-stained and reticulin-stained slides) were reviewed by two experienced liver pathologists (AB and VP). Each nodule was classified following the histological classification proposed by Park *et al.*[1] as RN, LGDN, HGDN, early HCC (eHCC), and HCC (Table S1). eHCC were distinguished from HCC according to their size being ≤2 cm. RN were distinguished from cirrhotic nodules by their size, ranging between 0.5 and 2 cm. Non-tumour livers were also evaluated. Non-tumour livers staged F0, F1, F2, and F3 according to the METAVIR system[22] were classified as noncirrhotic livers (NCL), whereas non-tumour livers staged F4 were classified as cirrhotic livers (CL). In the case of NCL, only nodules corresponding to HCC were included in the study.

Following the 2019 WHO grading system, HCC cases were classified by their differentiation (well, moderately, or poorly differentiated) and specific subtypes (steatohepatitic, clear cell, macrotrabecular massive, scirrhous, chromophobe, neutrophil-rich, and lymphocyte-rich) or as HCC not otherwise specified.[23]

Single-cell RNA sequencing

Single-cell RNA sequencing (RNAseq) datasets from Sharma *et al.*[24] and Xue *et al.*[25] were downloaded from the GepLiver collection of public liver transcriptomic datasets,[26] including raw counts and cell annotations. For each sample, we selected 'Endothelial cells' based on the GepLiver annotation, and transcriptomes from endothelial cells were summed gene-wise to obtain endothelial cell pseudobulks.[27] These pseudobulks were transformed using the vst function from the DESeq2 version 1.44.0 R package (Love Lab, Genetics and Biostatistics department, University of North Carolina, Chapel Hill, North Carolina, USA). FOLH1 (PSMA) gene expression was then visualized and tested across the HCC and non-tumour adjacent liver (ADJ_HCC) sample groups using Student's *t* test (Fig. 1).

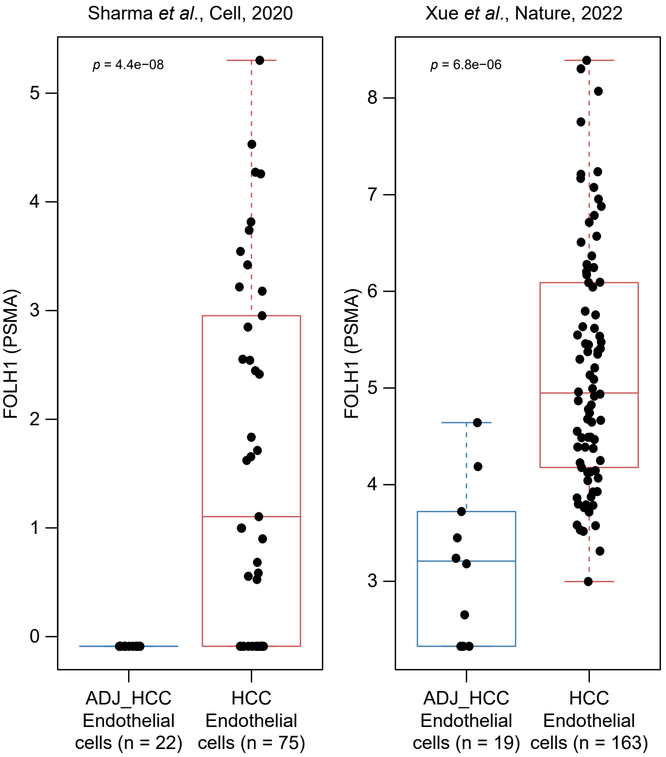

Fig. 1. FOLH1 (PSMA) gene expression in endothelial cells of the HCC and non-tumour adjacent liver (ADJ_HCC) samples from single-cell RNAseq datasets of Sharma *et al.*[24] and Xue *et al.*[25] Data are reported as VST-transformed counts (asymptotically equivalent to log_2). ADJ_HCC, non-tumour liver adjacent to the HCC; HCC, hepatocellular carcinoma.

Tissue microarray and immunohistochemistry

Tissue microarrays (TMAs) were constructed to preserve the available material and ensure good reproducibility between immunostainings.[3,28] One to four (1-mm) cores from each nodule and one core of non-tumour liver were used. Paraffin sections were stained for PSMA (EP192, ready to use; Vitro Master Diagnóstica, Granada, Spain), GS (glutamine synthetase-6, 1/2000; BD Biosciences, Franklin Lakes, NJ, USA), Glypican 3 (1G12, 1/100; Zytomed, Berlin, Germany), and HSP70 (2A4, 1/500; Abcam, Cambridge, UK) on a routine immunohistochemistry automate (Benchmark ultra, Ventana, Tuscon AZ, USA). HSP70 and Glypican 3 were considered positive when a staining of more than 5% of tumour cells was observed in at least one core, regardless of the intensity of the staining, as previously reported.[4,5] Positive PSMA staining was defined by the presence of staining in endothelial cells involving ≥5% of endothelial cells in at least one core, regardless of the intensity of the staining, as reported by Kmeid *et al.*[21] GS staining was considered positive when a diffuse (≥80%) and strong expression of tumour cells was observed (Fig. S1).

For 15 HCC resected cases, a paired preoperative tumour biopsy was available. For these cases, immunohistochemistry for PSMA, GS, Glypican 3, and HSP70 was performed on whole-slide sections of both the surgical specimen and the paired biopsy to compare staining patterns. PSMA staining on these whole slides was semi-quantitatively assessed based on the percentage of stained endothelial cells and the intensity of staining (weak, moderate, or strong).

Statistical analysis

Continuous variables are expressed as a range (mean; median), and categorical variables are expressed in the number of occurrences (%). The associations between PSMA immunostaining and clinicopathologic variables were assessed using the Pearson χ^2 test, Fisher's exact test, or the Mann–Whitney *U* test, when appropriate. Statistical analysis was performed using IBM SPSS Statistics for Windows version 29 (IBM Corp., Armonk, NY, USA) and GraphPad Prism version 10.2.2 (GraphPad Software, Boston, MA, USA). For each panel of markers, defined as a combination of one, two, three, or four different markers (a total of 15 possible combinations), the sensitivity, specificity, positive (PPV) and negative (NPV) predictive values, and accuracy were calculated using every possible minimal number of positive immunostaining as a decision threshold (*e.g.* for a panel of three markers, thresholds of one, two, and three positive markers are tested). A total of 32 immunostaining conditions (either individual markers or combinations) and threshold settings were evaluated for their diagnostic performance in the test cohort. The best-performing conditions were then assessed in the validation cohort. A *p* value <0.05 was considered statistically significant.

Results

PSMA gene expression in endothelial cells

Two single-cell RNAseq datasets (from Sharma *et al.*[24] [endothelial cells from non-tumour liver adjacent to the HCC, n = 22 cases; HCC endothelial cells, n = 75 cases] and from Xue *et al.*[25] [endothelial cells from non-tumour liver adjacent to the HCC, n = 19 cases; HCC endothelial cells, n = 163 cases]) were analysed, and both confirmed that FOLH1 (PSMA) gene expression was almost restricted to endothelial cells, with no or weak expression in HCC hepatocytes (data not shown) and was higher in HCC endothelial cells than in non-tumour adjacent liver endothelial cells (Fig. 1).

Study population

The clinicopathological characteristics of the patients included in the two cohorts are reported in Table 1. In total, 95/188 patients (51%) presented cirrhosis in the test cohort and 46/48 in the validation cohort (96%; *p* <0.001). They were predominantly males (n = 150/188 [80%] and n = 42/48 [88%], respectively), and the median age was 62 years in both cohorts. The main aetiologies of chronic liver diseases were HCV (n = 66/188 [35%]) in the test cohort and excessive alcohol

Table 1. Clinicopathological features of the population.

Features	Test cohort (n = 188) (%)	Validation cohort (n = 48) (%)	*p* value
Age (years), range (mean; median)	21–89 (61; 62)	38–73 (61; 62)	0.900
Sex			
Male	150 (80)	42 (88)	0.309
Female	38 (20)	6 (12)	0.309
Number of nodules per patient, range (mean; median)	1–25 (1.7; 1)	1–3 (1.8; 2)	<0.001
Nodules size (cm), range (mean; median)	0.3–25 (2.9; 1.2)	0.4–18 (1.8; 1.3)	0.309
Aetiology			
HBV	35 (19)	5 (10)	0.256
HCV	66 (35)	11 (23)	0.151
MS	60 (32)	18 (38)	0.574
Alcohol	43 (23)	25 (52)	<0.001
Others	13 (7)	2 (4)	0.742
Resection type			
Total hepatectomy	57 (30)	46 (96)	<0.001
Partial resection	131 (70)	2 (4)	<0.001
Non-tumour liver fibrosis (METAVIR)			
F0	12 (6)	1 (2)	0.476
F1	21 (11)	1 (2)	0.055
F2	27 (14)	0 (0)	0.002
F3	33 (18)	0 (0)	0.001
F4	95 (51)	46 (96)	<0.001

Comparisons between the two cohorts were assessed using the Pearson χ^2 test, Fisher's exact test, or the Mann–Whitney *U* test, as appropriate. MS, metabolic syndrome.

intake in the validation cohort (n = 25/48 [52%]). The median number of nodules per patient was 1 (1–25) in the test cohort and 2 (1–3) in the validation cohort (*p* <0.001). The median size of the nodules was similar in the two cohorts (median: 1.2 *vs*. 1.3 cm, *p* = 0.309).

A total of 407 hepatocellular nodules were analysed, including 320 in the test cohort and 87 in the validation cohort. These comprised RN (n = 39/320 [12%] and n = 22/87 [25%]), LGDN (n = 38 [12%] and n = 16 [18%]), HGDN (n = 30 [9%] and n = 8 [9%]), eHCC (n = 107 [33%] and n = 24 [28%]), and HCC (n = 106 [33%] and n = 17 [20%]). In addition, 73 CL and 79 NCL were evaluated in the test cohort, and 36 CL and 1 NCL were evaluated in the validation cohort.

PSMA, Glypican 3, HSP70, and GS expression in hepatocellular nodules from the test cohort

Endothelial PSMA was expressed in 83% of HCC (n = 88/106) and 77% of eHCC (n = 82/107). In non-malignant nodules, PSMA expression was observed in 27% of HGDN (n = 8/30), 21% of LGDN (n = 8/38), and 18% of RN (n = 7/39). In non-tumour livers, PSMA expression was much lower, observed in only four cases of CL (5%) and one case of NCL (<1%).

HSP70 was expressed in 79% of HCC (n = 84/106), 42% of eHCC (n = 45/107), 7% of other nodules (7/107), and 15% of non-tumour livers (n = 23/152). Glypican 3 was expressed in 65% of HCC (n = 69/106), 40% of eHCC (n = 43/107), 3% of other nodules (3/107), and 2% of non-tumour livers (n = 3/152). A diffuse and strong expression of GS was observed in 29% of HCC (n = 31/106), 21% of eHCC (n = 22/107), <1% of other nodules (n = 1/107), and <1% of non-tumour livers (n = 1/152) (Fig. 2 and Table S2).

Among HCC and eHCC cases (n = 213), PSMA identified 29 cases (14%) that came up negative for the other markers, whereas HSP70 identified only five cases (2%), Glypican 3 identified three cases (1%), and GS identified two cases (1%) that were not identified by the other three (Fig. S2).

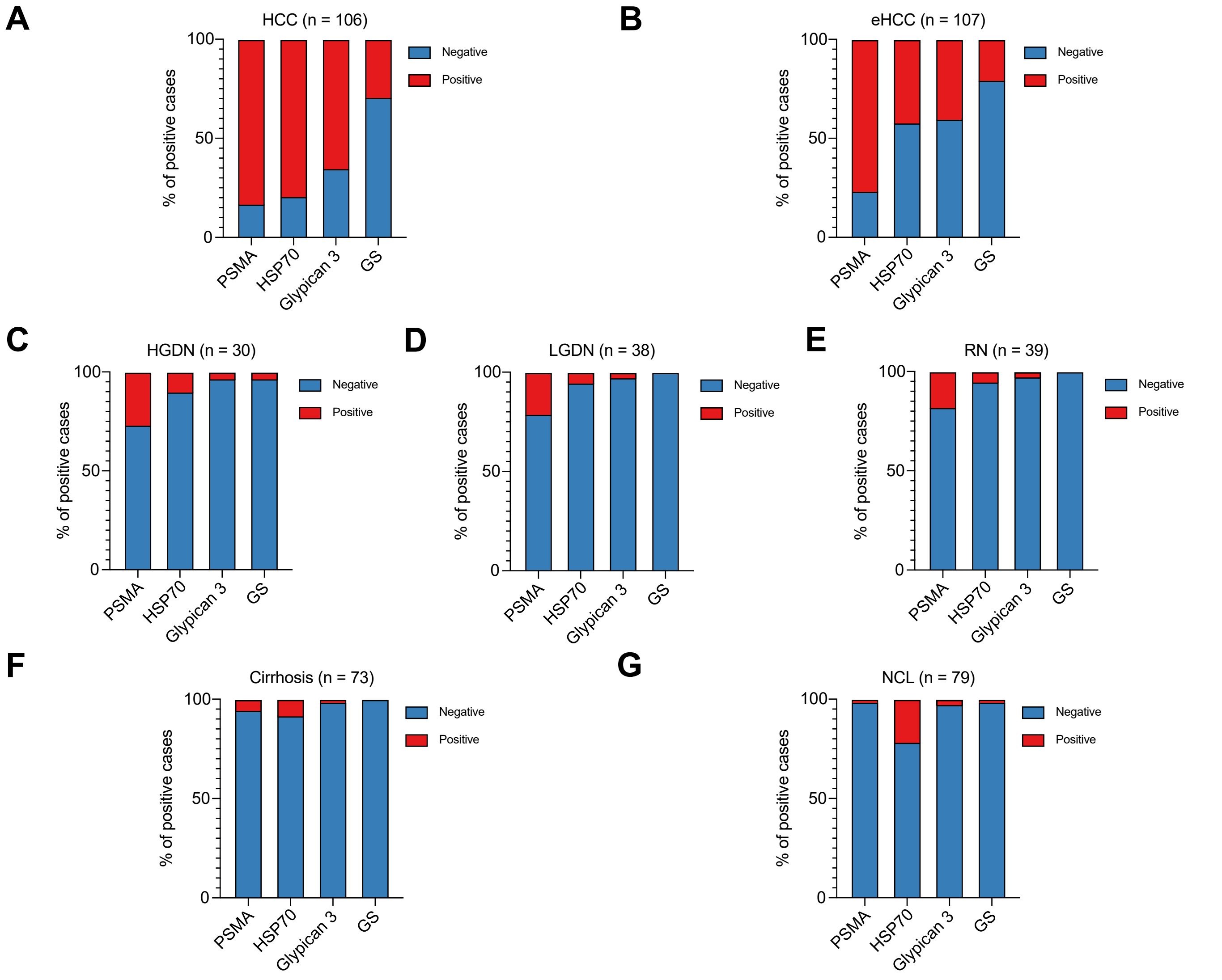

Fig. 2. Expression of PSMA, HSP70, Glypican 3, and GS in different types of nodules, cirrhosis and NCL in the test cohort. Expression in (A) HCC, (B) eHCC, (C) HGDN, (D) LGDN, (E) RN, (F) cirrhosis, and (G) NCL.

For PSMA immunostaining, among nodules with multiple cores analysed (n = 323), staining was identical across all cores in 245 cases (76%). In the remaining cases (n = 78/323, 24%), 55 (71%) showed PSMA staining in at least 50% of the cores from the same nodule.

Expression of PSMA, Glypican 3, HSP70, and GS in hepatocellular nodules on whole-slide sections from paired surgical specimens and biopsies

There was a complete concordance in PSMA expression among the 15 pairs of HCC biopsies (n = 15/15) and surgical specimens (n = 15/15) (median staining: 40% *vs*. 60%, p = 0.325; strong intensity of staining: 10/15 cases *vs*. 12/15 cases, p = 0.334). However, for the other staining, we observed differences between the paired samples. For Glypican 3, we observed positive staining for 8/15 surgical specimens and 6/15 biopsies, showing a reproducibility of 87%. For HSP70, we observed positive staining for 11/15 surgical specimens and 7/15 biopsies, showing a reproducibility of 73%. For GS, we observed positive staining for 5/15 surgical specimens and 8/15 biopsies, showing a reproducibility of 80%.

Whole-slide sections of NTL tissue paired with the 15 HCC were also assessed for PSMA staining, confirming that staining was weak or absent in endothelial cells in all cases (≤5% of endothelial cells), regardless of the stage of fibrosis in the underlying liver. When present, the staining showed low intensity, mostly in zone 2 of the lobule (Fig. 3 and Fig. S3).

HCC diagnosis performance of PSMA compared with other markers in the test cohort

The performances of each marker alone or in combination for HCC diagnosis are summarized in Table 2 for the whole cohort and in Table 3 for small nodules (≤2 cm). The sensitivity and specificity of PSMA alone for HCC diagnosis were 0.80 and 0.89, respectively, and its accuracy was 0.85. The sensitivity and the specificity of the Glypican 3–HSP70–GS (≥2 positive markers) combination for HCC diagnosis were 0.47 and 0.99, respectively, and its accuracy was 0.76. Adding PSMA to this combination increased sensitivity and accuracy to 0.73 and 0.88, respectively.

In summary, within the different tested combinations, PSMA alone showed the best sensitivity (0.80) and the best NPV (0.84). The best specificity (0.99) was observed with GS alone, or the combination of Glypican 3, HSP70, and GS (at least two positive markers), or the combination of PSMA, Glypican 3, HSP70, and GS (at least two positive markers). The best PPV (0.99) and best accuracy (0.88) were observed with the combination of PSMA, Glypican 3, HSP70, and GS (at least two positive markers) (Fig. 4B and Table S3).

In small nodules (≤2 cm), the sensitivity and specificity of PSMA alone for HCC diagnosis were 0.77 and 0.85, respectively, and its accuracy was 0.82. The sensitivity and specificity of the Glypican 3–HSP70–GS (≥2 positive markers) combination for HCC diagnosis were 0.30 and 0.99, respectively, and its accuracy was 0.73. Adding PSMA to this combination increased sensitivity and accuracy to 0.57 and 0.83, respectively.

Within the different tested combinations in small nodules (≤2 cm), PSMA alone showed the best sensitivity (0.77) and best NPV (0.86), compared with combinations of markers or other markers alone for diagnosing HCC. The best specificity (0.99) was observed with GS alone, or the combination of Glypican 3, HSP70, and GS (at least two positive markers), or the combination of PSMA, Glypican 3, HSP70, and GS (at least two positive markers). The best PPV (0.97) and accuracy (0.83) were observed with the combination of PSMA, Glypican 3, HSP70, and GS (at least two positive markers) (Fig. 4C and Table S4).

Table 2. Performance of PSMA, Glypican 3, HSP70, and GS alone or in combination for diagnosing HCC in the test cohort (n = 472, including 320 hepatocellular nodules and 152 non-tumour livers) and in the validation cohort (n = 124, including 87 hepatocellular nodules and 37 non-tumour livers).

Markers	Minimum positive markers required	Test cohort (n = 472)					Validation cohort (n = 124)				
		Se	Spe	PPV	NPV	Accuracy	Se	Spe	PPV	NPV	Accuracy
PSMA	1	**0.80**	0.89	0.86	**0.84**	0.85	**0.95**	0.77	0.67	**0.97**	0.83
Glypican 3	1	0.53	0.98	0.95	0.71	0.77	0.41	0.99	0.94	0.77	0.80
HSP70	1	0.61	0.88	0.81	0.73	0.76	0.76	0.53	0.44	0.81	0.60
GS	1	0.25	**0.99**	0.96	0.62	0.66	0.29	**1**	**1**	0.74	0.77
Glypican 3, HSP70, GS	2	0.47	**0.99**	0.98	0.69	0.76	0.41	0.99	0.94	0.77	0.80
PSMA, Glypican 3, HSP70, GS	2	0.73	**0.99**	**0.99**	0.82	**0.88**	0.85	0.87	0.76	0.92	**0.86**

Best performance for each metric is highlighted in bold. GS, glutamine synthetase; HSP70, heat shock protein 70; NPV, negative predictive value; PPV, positive predictive value; PSMA, prostate-specific membrane antigen; Se, sensitivity; Spe, specificity.

Table 3. Immunostaining performances of PSMA, Glypican 3, HSP70, and GS alone or in combination for diagnosing HCC in small nodules (≤2 cm).

Markers	Minimum positive markers required	Test cohort (n = 287)					Validation cohort (n = 106)				
		Se	Spe	PPV	NPV	Accuracy	Se	Spe	PPV	NPV	Accuracy
PSMA	1	**0.77**	0.85	0.75	**0.86**	0.82	**0.92**	0.77	0.54	**0.97**	0.80
Glypican 3	1	0.40	0.98	0.91	0.73	0.76	0.38	0.99	0.90	0.84	**0.85**
HSP70	1	0.42	0.93	0.78	0.73	0.74	0.67	0.54	0.30	0.85	0.57
GS	1	0.21	**0.99**	0.96	0.68	0.70	0.33	**1**	**1**	0.84	**0.85**
Glypican 3, HSP70, GS	2	0.30	**0.99**	0.94	0.70	0.73	0.38	0.99	0.90	0.84	**0.85**
PSMA, Glypican 3, HSP70, GS	2	0.57	**0.99**	**0.97**	0.79	**0.83**	0.79	0.87	0.63	0.93	**0.85**

Best performance for each metric is highlighted in bold. GS, glutamine synthetase; HSP70, heat shock protein 70; NPV, negative predictive value; PPV, positive predictive value; PSMA, prostate-specific membrane antigen; Se, sensitivity; Spe, specificity.

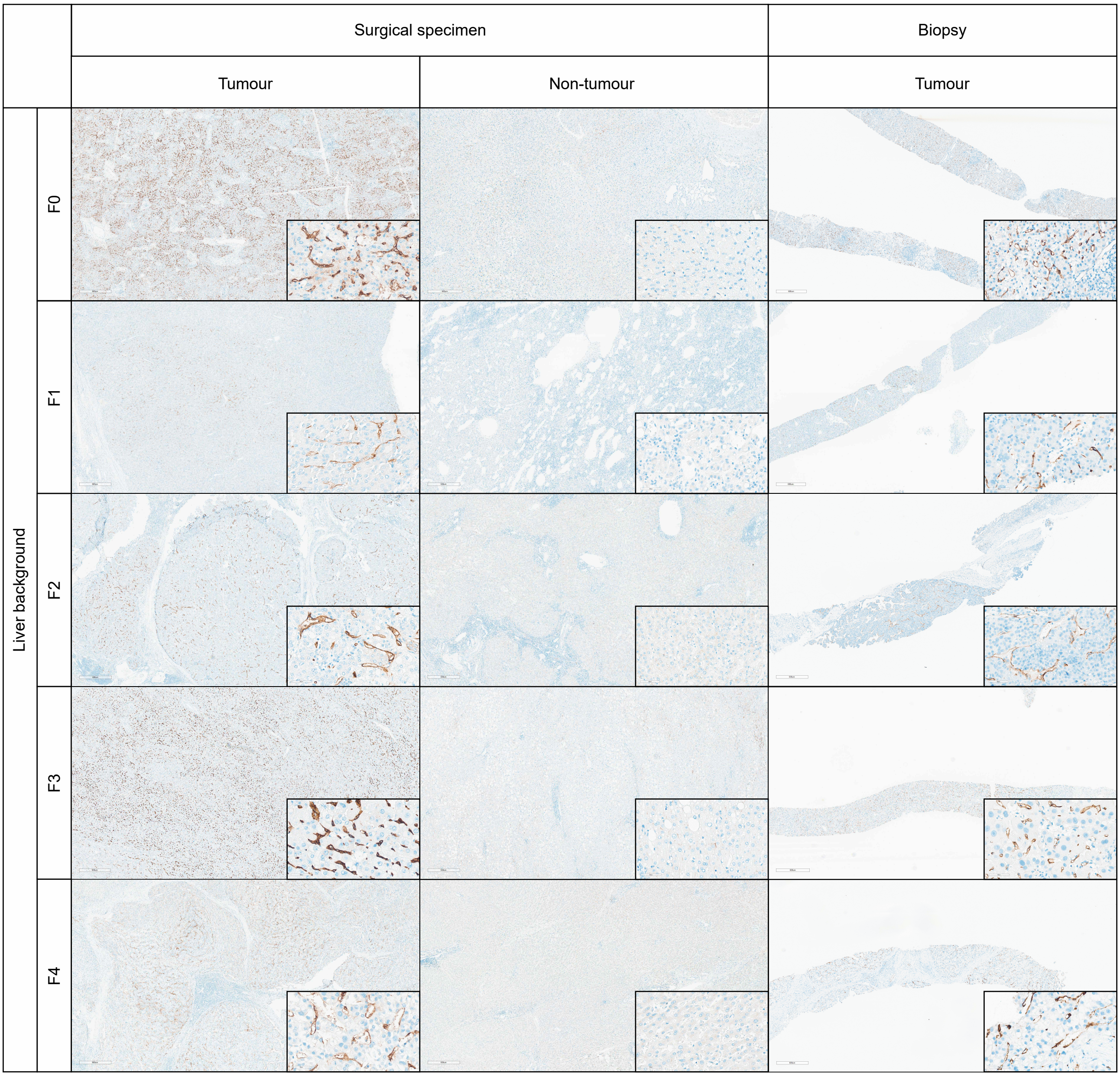

Fig. 3. Examples of PSMA expression in whole-slide images of paired surgical specimens (tumour and non-tumour livers) and biopsies from HCC cases, categorized by different stages of background liver fibrosis. HCC, hepatocellular carcinoma; PSMA, prostate-specific membrane antigen.

PSMA expression and clinicopathologic associations in the test cohort

We compared clinicopathological features according to the expression of PSMA in HCC cases (Table 4). The clear cell subtype was significantly more frequently observed in PSMA-negative cases compared with PSMA-positive cases (5 cases [12%] *vs*. 1 case [1%], p = 0.001). Microscopic vascular invasion was significantly more present in PSMA-positive cases compared with negative cases (65 cases [38%] *vs*. 7 cases [16%], p = 0.009). PSMA-negative cases corresponded mostly to well-differentiated HCC (29 cases [67%] *vs*. 67 cases [39%], p = 0.002), whereas PSMA-positive cases were mostly classified as moderately differentiated HCC (92 cases [54%] *vs*. 14 cases [33%], p = 0.019).

Validation of HCC diagnosis performance of PSMA compared with other markers in the external cohort

In the validation cohort, PSMA positivity was observed in 100% of HCC (n = 17/17) and 92% of early HCC (n = 22/24). The diagnostic performance of PSMA for HCC was maintained in this cohort. PSMA alone achieved a sensitivity of 0.95 and a specificity of 0.77, with an overall accuracy of 0.83. The combination of Glypican 3, HSP70, and GS (defined as ≥2 positive markers) demonstrated a sensitivity of 0.41, a specificity of 0.99, and an accuracy of 0.80. Adding PSMA to this panel significantly improved sensitivity and accuracy (0.85 and 0.86, respectively) while maintaining high specificity (0.87) (Table 2, Fig. 4E, and Table S5).

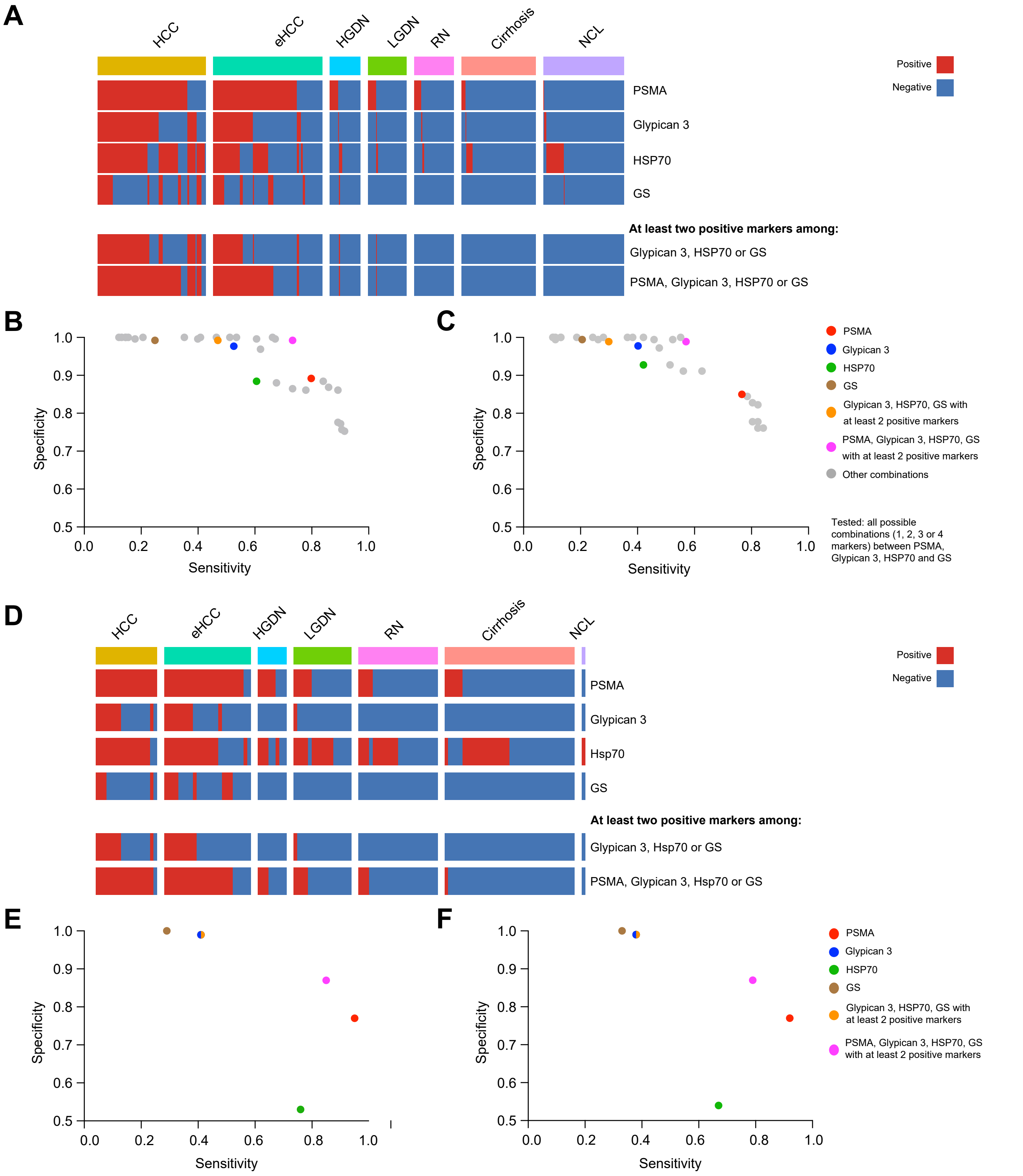

Fig. 4. Expression and performance of PSMA, Glypican 3, HSP70, and GS markers alone or in combination for the diagnosis of HCC in the test and validation cohorts. Expression and performance of markers alone or in combination in the test cohort (A-C)Expression and performance of markers alone or in combination in the validation cohort (D-F).

Table 4. Clinicopathological features according to the expression of PSMA in HCC cases of the test cohort (n = 213).

Features	PSMA+ (%) (n = 170)	PSMA- (%) (n = 43)	*p* value
MS	53 (31)	9 (21)	0.257
Alcohol	40 (24)	13 (30)	0.477
HCV	65 (38)	11 (26)	0.171
HBV	31 (18)	7 (16)	0.939
Others	9 (5)	4 (9)	0.302
Well-limited tumour	122 (72)	30 (70)	0.944
Mean size (cm)	4 (0.5–25)	3 (0.3–14)	0.067
HCC subtypes			
NOS HCC	114 (67)	28 (65)	0.952
SH HCC	45 (26)	10 (23)	0.814
MTM HCC	7 (4)	0 (0)	0.349
Clear cells	1 (1)	5 (12)	**0.001**
Vascular invasion			
Microscopic	65 (38)	7 (16)	**0.009**
Macroscopic	14 (8)	0 (0)	0.079
Satellite nodules	25 (15)	4 (9)	0.459
HCC differentiation			
Well differentiated	67 (39)	29 (67)	**0.002**
Moderately differentiated	92 (54)	14 (33)	**0.019**
Poorly differentiated	11 (6)	0 (0)	0.126
AFP concentration (ng/ml), range (mean; median)	1–31,546 (1,308; 10)	2–12,747 (548; 5)	0.132
BCLC score			
0	20 (12)	3 (7)	0.582
A	104 (61)	24 (56)	0.640
B	45 (26)	16 (37)	0.229
C	1 (1)	0 (0)	1
Typical aspect on imagery	119 (70)	24 (56)	0.112

The associations between PSMA immunostaining and clinicopathologic variables were assessed using the Pearson χ^2 test, Fisher's exact test, or the Mann–Whitney *U* test, when appropriate. Significant *p* values (<0.05) are highlighted in bold. AFP, alpha-foetoprotein; BCLC, Barcelona Clinic Liver Cancer; HCC, hepatocellular carcinoma; MS, metabolic syndrome; MTM, macro-trabecular massive; NOS, not otherwise specified; SH, steatohepatitic.

In small nodules (≤2 cm), PSMA alone showed a sensitivity of 0.92, a specificity of 0.77, and an accuracy of 0.80. The Glypican 3–HSP70–GS combination had a sensitivity of 0.38, a specificity of 0.99, and an accuracy of 0.85. The addition of PSMA to this panel enhanced sensitivity (0.79) while preserving high specificity (0.87) (Table 3 and Fig. 4F).

Discussion

In this study, we assessed the diagnostic value of PSMA for identifying HCC among two independent series of 320 and 87 nodules developed in the cirrhotic and non-cirrhotic backgrounds, respectively. PSMA positivity was observed in 86% of HCC and 80% of early HCC in the whole cohort. This percentage was consistent with data previously reported in the literature.[21,29–31]

To our knowledge, this study is the first to compare the diagnostic performance of PSMA with the markers usually used in routine practice for distinguishing precursor lesions from HCC, namely Glypican 3, HSP70, and GS.[4,5,15] Interestingly, Kmeid *et al.*[21] have compared PSMA with CD34, a marker of relatively limited interest in this diagnostic task,[32] and have already shown that PSMA was more accurate than CD34 (95.5% *vs*. 69.7%) in distinguishing grade 1 HCC from hepatocellular adenoma and HGDN. Our results showed that PSMA outperformed the current combination of Glypican 3, HSP70, and GS in most of the performance metrics used in this study in the two cohorts, particularly in the sensitivity, 0.95 *vs*. 0.41, respectively, for HCC diagnosis in the validation cohort. The sensitivity of the combination of Glypican 3, HSP70, and GS was lower than that described in the literature (between 0.58 and 0.89), whereas we obtained a similar specificity, approximately 100%.[4,5,15,33] This difference could be explained by the composition of our series, with a higher proportion of early HCC (52%) than in previous studies.[4,5,15,33] However, PSMA showed reduced specificity (0.89 in the test cohort and 0.77 in the validation cohort) compared with the other markers, with a decrease ranging from 9% to 23% depending on the marker and the cohort. Therefore, the best overall performance across all evaluated metrics in the two cohorts was obtained by adding PSMA to the combination of Glypican 3, HSP70, and GS, with at least two positive markers. Nevertheless, it requires sufficient material to perform all four stainings. Thus, in the context of sparse material, such as a biopsy, it may be advisable to only perform the PSMA immunostaining. Moreover, we have demonstrated that PSMA can be assessed in biopsies, showing similar results to those obtained on surgical specimens.

As performed in several studies, we considered as positive a PSMA staining in capillarised sinusoidal/tumour-associated vessel staining involving ≥5% of the tumour area,[21,29] making assessment relatively simple and therefore easily reproducible.

In addition to its diagnostic performance, several studies have suggested its prognostic value in the context of HCC.[29,31,34] In particular, Jiao *et al.*[34] showed in 103 patients with HCC that vascular PSMA expression was associated with tumour stage, differentiation, Ki-67 proliferation index, and lymph node metastasis and that higher expression was correlated with shorter overall survival. Even if this was not the objective of our study, we compared some histological prognostic factors according to PSMA expression. Similar to Jiao *et al.*,[34] we observed an association between PSMA expression and HCC differentiation (Table 4). Interestingly, we also assessed PSMA expression according to HCC subtypes and

observed lower expression in clear cell HCC, which is considered a subtype of good prognosis.[35] We also observed that PSMA expression was correlated with microscopic vascular invasion, which is a factor present in more aggressive tumours.[36]

Finally, as previously shown in prostatic cancer,[17,18] studies evaluating PSMA in HCC by both immunohistochemistry and imaging have shown a close correlation between intratumoural PSMA microvessel staining and gallium-68-PSMA uptake on positron emission tomography (PET).[30,37] Thus, this opens new diagnostic and therapeutic opportunities, similar to those in prostate cancer, with tumours with high PSMA PET avidity potentially being eligible for PSMA-targeted radionuclide therapy.[18,38]

The main limitations of this study include its retrospective design and the assessment of staining mainly on TMA from surgical specimens. The assessment of PSMA staining could be interesting in a larger series of biopsies. However, according to the international guidelines, the diagnosis of hepatocellular nodules in patients with cirrhosis is routinely based on imaging, limiting the number of biopsies performed.[39] Another limitation is that not all nodules originated from different patients, meaning some share the same genetic background. In addition, the test cohort includes a high proportion of HCV-related cases (35%), which is not representative of the general population. Finally, we did not assess PSMA expression in other types of primary malignant liver tumours, such as cholangiocarcinoma or combined hepatocellular–cholangiocarcinoma. However, Chen *et al.*[40] showed that although PSMA expression is present in both HCC and cholangiocarcinoma, it is significantly higher in HCC. Nonetheless, its presence in both tumour types may limit its specificity, particularly in cases involving combined tumours or poorly differentiated lesions. Further prospective studies with larger cohorts are needed to validate the diagnostic accuracy of PSMA in these challenging contexts.

In conclusion, PSMA demonstrated good performance in diagnosing HCC, superior in two independent series to the combination of three routinely used markers (Glypican 3, HSP70, and GS). It could be particularly useful in clinical practice for differentiating difficult-to-classify hepatocellular nodules at a lower cost, using a technique readily available in all pathology laboratories, thus ensuring its possible application. When the material is sparse, using PSMA alone could be recommended, whereas when sufficient material is available, adding PSMA to Glypican 3, HSP70, and GS may be advised, as this combination has shown the best accuracy for HCC diagnosis.

Affiliations

[1]*Université Paris Cité, Centre de Recherche sur l'Inflammation (CRI), INSERM, U1149, CNRS, ERL 8252, Paris, France;* [2]*AP-HP.GHU Paris Saclay, Department of Pathology, Bicêtre Hospital, Le Kremlin-Bicêtre, France;* [3]*AP-HP.Nord, Department of Pathology, RHU OPERANDI, FHU MOSAIC[2], Siric InsiTu, DMU DREAM, Beaujon Hospital, Clichy, France;* [4]*Department of Nuclear Medicine, RHU OPERANDI, University Hospital of Nantes, Nantes, France;* [5]*AP-HP.Nord, Liver Cancer Unit, DMU DIGEST, Beaujon Hospital, Clichy, France;* [6]*AP-HP.Nord, Department of HPB Surgery and Liver Transplantation, Beaujon Hospital, Université Paris Cité, Clichy, France;* [7]*AP-HP.Nord, Department of Nuclear Medicine, RHU OPERANDI, FHU MOSAIC, DMU DREAM, Beaujon Hospital, Clichy, France;* [8]*AP-HP. Nord, Department of Radiology, RHU OPERANDI, FHU MOSAIC, DMU DREAM, Beaujon Hospital, Clichy, France*

Abbreviations

CL, cirrhotic liver; eHCC, early HCC; GS, glutamine synthetase; HCC, hepatocellular carcinoma; HCC-HGDN, HCC developed in HGDN; HGDN, high-grade dysplastic nodules; HSP70, heat shock protein 70; LGDN, low-grade dysplastic nodules; NCL, non-cirrhotic livers; NOS, not otherwise specified; NPV, negative predictive value; NTL, non-tumour livers; PPV, positive predictive value; PSMA: prostate-specific membrane antigen; RN, regenerative nodules; RNAseq, RNA sequencing; Se, sensitivity; Spe, specificity; TMA, tissue microarray.

Financial support

This work, performed under the RHU OPERANDI, was supported in part by the French National Research Agency (Agence Nationale de la Recherche, ANR) as its 3rd PIA, integrated to France 2030 plan under reference ANR-21-RHUS-0012.

Conflicts of interest

The authors declare no conflict of interest.

Please refer to the accompanying ICMJE disclosure forms for further details.

Authors' contributions

Study concept and design: AB, JC, CB. Acquisition of data: KV, AB, SL, HC, MA, JC, ALB, CG, CB. Analysis and interpretation of data: KV, EB, AB, JC, VP, VV. Drafting of the manuscript: KV, AB, JC. Critical revision of the manuscript for important intellectual content: RN, EB, ML, MB, RL, VV, VP. Statistical analysis: KV, RN, AB. Obtained funding: VV, JC. Study supervision: AB, JC.

Data availability

The datasets used and/or analysed during the current study are available from the corresponding author upon reasonable request.

Patient consent statement and ethics approval statement

Written consent was obtained from all patients as required by French legislation. This study was approved by the local ethics committee (CER PARIS NORD no. 2020-015-IRB 00006477).

Acknowledgements

Figures were made using BioRender, GraphPad Prism Version 10.4.1 (532), and Inkscape 1.4 (e7c3feb1, 2024-10-09).

Supplementary data

Supplementary data to this article can be found online at https://doi.org/10.1016/j.jhepr.2025.101542.

References

Author names in bold designate shared co-first authorship

[1] Park YN. Update on precursor and early lesions of hepatocellular carcinomas. Arch Pathol Lab Med 2011;135:704–715.

[2] Nault JC, Calderaro J, Di Tommaso L, et al. Telomerase reverse transcriptase promoter mutation is an early somatic genetic alteration in the transformation of premalignant nodules in hepatocellular carcinoma on cirrhosis. Hepatology 2014;60:1983–1992.

[3] Beaufrère A, Paisley S, Ba I, et al. Differential diagnosis of small hepatocellular nodules in cirrhosis: surrogate histological criteria of TERT promoter mutations. Histopathology 2024;84:473–481.

[4] Di Tommaso L, Franchi G, Park YN, et al. Diagnostic value of HSP70, glypican 3, and glutamine synthetase in hepatocellular nodules in cirrhosis. Hepatology 2007;45:725–734.

[5] Di Tommaso L, Destro A, Seok JY, et al. The application of markers (HSP70 GPC3 and GS) in liver biopsies is useful for detection of hepatocellular carcinoma. J Hepatol 2009;50:746–754.

[6] Helmbrecht K, Zeise E, Rensing L. Chaperones in cell cycle regulation and mitogenic signal transduction: a review. Cell Prolif 2000;33:341–365.

[7] Chuma M, Sakamoto M, Yamazaki K, et al. Expression profiling in multistage hepatocarcinogenesis: identification of HSP70 as a molecular marker of early hepatocellular carcinoma. Hepatology 2003;37:198–207.

[8] Häussinger D, Sies H, Gerok W. Functional hepatocyte heterogeneity in ammonia metabolism. The intercellular glutamine cycle. J Hepatol 1985;1:3–14.

[9] **Rebouissou S**, **Franconi A**, Calderaro J, et al. Genotype-phenotype correlation of CTNNB1 mutations reveals different ß-catenin activity associated with liver tumor progression. Hepatology 2016;64:2047–2061.

[10] Capurro M, Wanless IR, Sherman M, et al. Glypican-3: a novel serum and histochemical marker for hepatocellular carcinoma. Gastroenterology 2003;125:89–97.

[11] Zhu ZW, Friess H, Wang L, et al. Enhanced glypican-3 expression differentiates the majority of hepatocellular carcinomas from benign hepatic disorders. Gut 2001;48:558–564.

[12] Sung YK, Hwang SY, Park MK, et al. Glypican-3 is overexpressed in human hepatocellular carcinoma. Cancer Sci 2003;94:259–262.

[13] Yamauchi N, Watanabe A, Hishinuma M, et al. The glypican 3 oncofetal protein is a promising diagnostic marker for hepatocellular carcinoma. Mod Pathol 2005;18:1591–1598.

[14] Di Tommaso L, Destro A, Fabbris V, et al. Diagnostic accuracy of clathrin heavy chain staining in a marker panel for the diagnosis of small hepatocellular carcinoma. Hepatology 2011;53:1549–1557.

[15] Tremosini S, Forner A, Boix L, et al. Prospective validation of an immunohistochemical panel (glypican 3, heat shock protein 70 and glutamine synthetase) in liver biopsies for diagnosis of very early hepatocellular carcinoma. Gut 2012;61:1481–1487.

[16] Troyer JK, Beckett ML, Wright GL. Detection and characterization of the prostate-specific membrane antigen (PSMA) in tissue extracts and body fluids. Int J Cancer 1995;62:552–558.

[17] Weineisen M, Schottelius M, Simecek J, et al. ^{68}Ga- and ^{177}Lu-labeled PSMA I&T: optimization of a PSMA-targeted theranostic concept and first proof-of-concept human studies. J Nucl Med 2015;56:1169–1176.

[18] Zhang H, Koumna S, Pouliot F, et al. PSMA theranostics: current landscape and future outlook. Cancers 2021;13:4023.

[19] Chang SS, Reuter VE, Heston WD, et al. Five different anti-prostate-specific membrane antigen (PSMA) antibodies confirm PSMA expression in tumor-associated neovasculature. Cancer Res 1999;59:3192–3198.

[20] Haffner MC, Kronberger IE, Ross JS, et al. Prostate-specific membrane antigen expression in the neovasculature of gastric and colorectal cancers. Hum Pathol 2009;40:1754–1761.

[21] Kmeid M, Park YN, Chung T, et al. PSMA immunohistochemistry in hepatic neoplasms: a promising diagnostic marker with potential theranostic applications. Am J Surg Pathol 2022;46:1688–1699.

[22] Bedossa P, Poynard T. An algorithm for the grading of activity in chronic hepatitis C. The METAVIR Cooperative Study Group. Hepatology 1996;24:289–293.

[23] WHO Classification of Tumours Editorial Board. Digestive system tumours. 1. 5th ed. Lyon: International Agency for Research on Cancer; 2019.

[24] Sharma A, Seow JJW, Dutertre C-A, et al. Onco-fetal reprogramming of endothelial cells drives immunosuppressive macrophages in hepatocellular carcinoma. Cell 2020;183:377–394.e21.

[25] **Xue R**, **Zhang Q**, **Cao Q**, et al. Liver tumour immune microenvironment subtypes and neutrophil heterogeneity. Nature 2022;612:141–147.

[26] **Li Z**, **Zhang H**, Li Q, et al. GepLiver: an integrative liver expression atlas spanning developmental stages and liver disease phases. Sci Data 2023;10:376.

[27] Murphy AE, Skene NG. A balanced measure shows superior performance of pseudobulk methods in single-cell RNA-sequencing analysis. Nat Commun 2022;13:7851.

[28] Trapani L, Beaufrère A, Hobeika C, et al. Pathological overview of steatohepatitic hepatocellular carcinoma in a surgical series. Histopathology 2023;83:526–537.

[29] Li RX, Hao Y, Ettel M. Expression of PSMA in tumor-associated vasculature predicts poorer survival in patients with hepatocellular carcinoma and is likely associated with PD-L1. Int J Surg Pathol 2024;32:1248–1255.

[30] Thompson SM, Suman G, Torbenson MS, et al. PSMA as a theranostic target in hepatocellular carcinoma: immunohistochemistry and 68 Ga-PSMA-11 PET using cyclotron-produced 68 Ga. Hepatol Commun 2022;6:1172–1185.

[31] Tolkach Y, Goltz D, Kremer A, et al. Prostate-specific membrane antigen expression in hepatocellular carcinoma: potential use for prognosis and diagnostic imaging. Oncotarget 2019;10:4149–4160.

[32] Park YN, Yang CP, Fernandez GJ, et al. Neoangiogenesis and sinusoidal "capillarization" in dysplastic nodules of the liver. Am J Surg Pathol 1998;22:656–662.

[33] Vasuri F, Malvi D, Bonora S, et al. From large to small: the immunohistochemical panel in the diagnosis of early hepatocellular carcinoma. Histopathology 2018;72:414–422.

[34] Jiao D, Li Y, Yang F, et al. Expression of prostate-specific membrane antigen in tumor-associated vasculature predicts poor prognosis in hepatocellular carcinoma. Clin Transl Gastroenterol 2019;10:1–7.

[35] Deng Y, Zhu S, Yan W, et al. Influence of clear cell carcinoma on the post-hepatectomy prognosis of patients with hepatocellular carcinoma. Future Oncol 2022;18:543–552.

[36] Beaufrère A, Caruso S, Calderaro J, et al. Gene expression signature as a surrogate marker of microvascular invasion on routine hepatocellular carcinoma biopsies. J Hepatol 2022;76:343–352.

[37] Kesler M, Levine C, Hershkovitz D, et al. ^{68}Ga-PSMA is a novel PET-CT tracer for imaging of hepatocellular carcinoma: a prospective pilot study. J Nucl Med 2019;60:185–191.

[38] Kratochwil C, Fendler WP, Eiber M, et al. EANM procedure guidelines for radionuclide therapy with ^{177}Lu-labelled PSMA-ligands (^{177}Lu-PSMA-RLT). Eur J Nucl Med Mol Imaging 2019;46:2536–2544.

[39] European Association for the Study of the Liver. EASL Clinical Practice Guidelines: management of hepatocellular carcinoma. J Hepatol 2018;69:182–236.

[40] Chen L-X, Zou S-J, Li D, et al. Prostate-specific membrane antigen expression in hepatocellular carcinoma, cholangiocarcinoma, and liver cirrhosis. World J Gastroenterol 2020;26:7664–7678.

Keywords: PSMA; Diagnostic biomarker; Immunohistochemistry; Hepatocellular carcinoma; Dysplastic nodule; Endothelial cells.
Received 19 February 2025; received in revised form 23 July 2025; accepted 25 July 2025; Available online 31 July 2025

Journal of Hepatology, Volume *7*

Supplemental information

PSMA immunohistochemistry as a diagnostic biomarker of hepatocellular carcinoma

Killian Véron, Etienne Becht, Astrid Laurent-Bellue, Rémy Nicolle, Miguel Albuquerque, Samira Laouirem, Hélène Cazier, Clément Bailly, Mohamed Bouattour, Mickaël Lesurtel, Catherine Guettier, Rachida Lebtahi, Valérie Vilgrain, Valérie Paradis, Jérôme Cros, and Aurélie Beaufrère

PSMA immunohistochemistry as a diagnostic biomarker of hepatocellular carcinoma

Killian Véron, Etienne Becht, Astrid Laurent-Bellue, Rémy Nicolle, Miguel Albuquerque, Samira Laouirem, Hélène Cazier, Clément Bailly, Mohamed Bouattour, Mickaël Lesurtel, Catherine Guettier, Rachida Lebtahi, Valérie Vilgrain, Valérie Paradis, Jérôme Cros, Aurélie Beaufrère

Table of contents

Table S1 2

Table S2 3

Table S3. 4

Table S4. 6

Table S5. 8

Fig. S1. 9

Fig. S2. 10

Fig. S3. 11

Table S1. List of morphological criteria assessed for each nodule and their definition.

Morphological criteria	Definition
Small-cell change	Cluster of small cells with decreased nucleo-cytoplasmic ratio
Large-cell change	Cluster of large cells with conserved nucleo-cytoplasmic ratio
Clone-like features (clear cells, fat)	Areas of clear cells and/or of cells containing fat
Increased cellular density	Nodule presented an increased cell density compared with extranodular cirrhotic liver
Presence of pseudoglands	Presence of pseudoglands or pseudorosettes architecture in the nodule
Loss of portal tracts	Absence of portal tracts in the nodule
Unpaired arteries	Presence of unpaired arteries in the nodule
Stromal invasion	Presence of tumour cells invading the fibrous tissue of portal tracts within or outside the nodule
Plate-thickening anomalies	Focal or diffuse increase of the thickness of the trabeculae (≥3 cells of thickness)
Alteration of the reticulin framework	Reduction or fragmentation or disappearance of the reticulinic framework

Table S2. Expression of PSMA, Glypican 3, HSP70, and GS across different nodule types and in the non-tumourous liver within the test cohort.

	PSMA+	Glypican 3+	HSP70+	GS+
Non-Cirrhotic Liver (n=79)	1 (1%)	2 (3%)	17 (22%)	1 (1%)
Cirrhosis (n=73)	4 (5%)	1 (1%)	6 (8%)	0 (0%)
RN (n=39)	7 (18%)	1 (3%)	2 (5%)	0 (0%)
LGDN (n=38)	8 (21%)	1 (3%)	2 (5%)	0 (0%)
HGDN (n=30)	8 (27%)	1 (3%)	3 (10%)	1 (3%)
eHCC (n=107)	82 (77%)	43 (40%)	45 (42%)	22 (21%)
HCC (n=106)	88 (83%)	69 (65%)	84 (79%)	31 (29%)

Table S3. Immunostaining performances of PSMA, Glypican 3, HSP70 and GS alone or in combination for diagnosing HCC within the test cohort (n=472).

Markers	Minimum of positive markers required	Se	Spe	PPV	NPV	Accuracy
PSMA	1	0.80	0.89	0.86	0.84	0.85
Glypican 3	1	0.53	0.98	0.95	0.71	0.77
HSP70	1	0.61	0.88	0.81	0.73	0.76
GS	1	0.25	0.99	0.96	0.62	0.66
PSMA, Glypican 3	1	0.86	0.87	0.84	0.88	0.81
PSMA, GS	1	0.84	0.88	0.86	0.87	0.86
PSMA, HSP70	1	0.89	0.78	0.77	0.90	0.83
Glypican 3, HSP70	1	0.73	0.86	0.82	0.80	0.81
Glypican 3, GS	1	0.62	0.97	0.94	0.76	0.81
HSP70, GS	1	0.68	0.88	0.82	0.77	0.79
PSMA, HSP70	2	0.51	1.00	1.00	0.71	0.78
PSMA, Glypican 3	2	0.46	1.00	1.00	0.69	0.71
Glypican 3, HSP70	2	0.40	1.00	0.99	0.67	0.73
PSMA, GS	2	0.21	1.00	1.00	0.61	0.64
HSP70, GS	2	0.18	1.00	0.97	0.60	0.63
Glypican 3, GS	2	0.15	1.00	1.00	0.59	0.62
PSMA, Glypican 3, GS	1	0.89	0.86	0.84	0.91	0.88
PSMA, HSP70, GS	1	0.90	0.77	0.76	0.90	0.83
PSMA, Glypican 3, HSP70	1	0.91	0.76	0.75	0.91	0.82

Glypican 3, HSP70, GS	1	0.78	0.86	0.82	0.83	0.82
PSMA, Glypican 3, HSP70	2	0.67	1.00	0.99	0.79	0.85
PSMA, HSP70, GS	2	0.61	1.00	0.99	0.75	0.82
PSMA, Glypican 3, GS	2	0.54	1.00	1.00	0.72	0.79
Glypican 3, HSP70, GS	2	0.47	0.99	0.98	0.69	0.76
PSMA, Glypican 3, HSP70	3	0.35	1.00	1.00	0.65	0.71
PSMA, Glypican 3, GS	3	0.15	1.00	1.00	0.59	0.61
PSMA, HSP70, GS	3	0.15	1.00	1.00	0.59	0.61
Glypican 3, HSP70, GS	3	0.13	1.00	1.00	0.58	0.61
PSMA, Glypican 3, HSP70, GS	1	0.92	0.75	0.75	0.92	0.83
PSMA, Glypican 3, HSP70, GS	2	0.73	0.99	0.99	0.82	0.88
PSMA, Glypican 3, HSP70, GS	3	0.41	1.00	1.00	0.67	0.73
PSMA, Glypican 3, HSP70, GS	4	0.12	1.00	1.00	0.58	0.60

Se, sensitivity, Sp, specificity, PPV, positive predictive value, NPV, negative predictive value

Table S4. Immunostaining performances of PSMA, Glypican 3, HSP70 and GS alone or in combination for diagnosing HCC in small nodules (≤2 cm) within the test cohort (n=213).

Markers	Minimum positive markers required	Se	Spe	PPV	NPV	Accuracy
PSMA	1	0.77	0.85	0.75	0.86	0.82
Glypican 3	1	0.40	0.98	0.91	0.73	0.76
HSP70	1	0.42	0.93	0.78	0.73	0.74
GS	1	0.21	0.99	0.96	0.68	0.70
PSMA, Glypican 3	1	0.80	0.83	0.74	0.88	0.82
PSMA, GS	1	0.79	0.84	0.75	0.87	0.82
PSMA, HSP70	1	0.80	0.78	0.68	0.87	0.79
Glypican 3, HSP70	1	0.56	0.91	0.79	0.78	0.78
Glypican 3, GS	1	0.48	0.97	0.91	0.76	0.79
HSP70, GS	1	0.51	0.93	0.81	0.76	0.77
PSMA, HSP70	2	0.38	1.00	1.00	0.73	0.77
PSMA, Glypican 3	2	0.36	1.00	1.00	0.73	0.76
Glypican 3, HSP70	2	0.26	0.99	0.97	0.69	0.72
PSMA, GS	2	0.19	1.00	1.00	0.67	0.70
Glypican 3, GS	2	0.13	1.00	1.00	0.66	0.68
HSP70, GS	2	0.11	0.99	0.92	0.65	0.67
PSMA, Glypican 3, GS	1	0.82	0.82	0.73	0.89	0.82
PSMA, HSP70, GS	1	0.82	0.78	0.69	0.88	0.79
PSMA, Glypican 3, HSP70	1	0.82	0.76	0.67	0.88	0.78

Glypican 3, HSP70, GS	1	0.63	0.91	0.81	0.80	0.80
PSMA, Glypican 3, HSP70	2	0.52	0.99	0.98	0.78	0.82
PSMA, HSP70, GS	2	0.46	0.99	0.98	0.76	0.79
PSMA, Glypican 3, GS	2	0.42	1.00	1.00	0.74	0.78
Glypican 3, HSP70, GS	2	0.30	0.99	0.94	0.70	0.73
PSMA, Glypican 3, HSP70	3	0.24	1.00	1.00	0.69	0.72
PSMA, Glypican 3, GS	3	0.13	1.00	1.00	0.66	0.68
PSMA, HSP70, GS	3	0.11	1.00	1.00	0.65	0.67
Glypican 3, HSP70, GS	3	0.10	1.00	1.00	0.65	0.67
PSMA, Glypican 3, HSP70, GS	1	0.84	0.76	0.68	0.89	0.79
PSMA, Glypican 3, HSP70, GS	2	0.57	0.99	0.97	0.79	0.83
PSMA, Glypican 3, HSP70, GS	3	0.28	1.00	1.00	0.70	0.73
PSMA, Glypican 3, HSP70, GS	4	0.10	1.00	1.00	0.65	0.67

Se: sensitivity, Sp: specificity, PPV: positive predictive value, NPV: negative predictive value

Table S5. Expression of PSMA, Glypican 3, HSP70, and GS across different nodule types and in the non-tumourous liver within the validation cohort.

	PSMA+	Glypican 3+	HSP70+	GS+
Non-Cirrhotic Liver (n=1)	0 (0%)	0 (0%)	1 (100%)	0 (0%)
Cirrhosis (n=36)	5 (14%)	0 (0%)	14 (39%)	0 (0%)
RN (n=22)	4 (18%)	0 (0%)	10 (45%)	0 (0%)
LGDN (n=16)	5 (31%)	1 (6%)	10 (63%)	0 (0%)
HGDN (n=8)	5 (63%)	0 (0%)	4 (50%)	0 (0%)
eHCC (n=24)	22 (92%)	9 (38%)	16 (67%)	8 (33%)
HCC (n=17)	17 (100%)	8 (47%)	15 (88%)	4 (24%)

Fig. S1. Examples of positive immunostainings in TMA HCC cases.

(A) Glutamine Synthetase: diffuse and intense expression of tumour cells, (B) Heat shock protein 70: nuclear expression of ≥5% of tumour cells, (C) Glypican 3: cytoplasmic and membranous expression of ≥5% of tumour cells, (D) Prostate Specific Membrane Antigen: expression of ≥5% of endothelial cells.

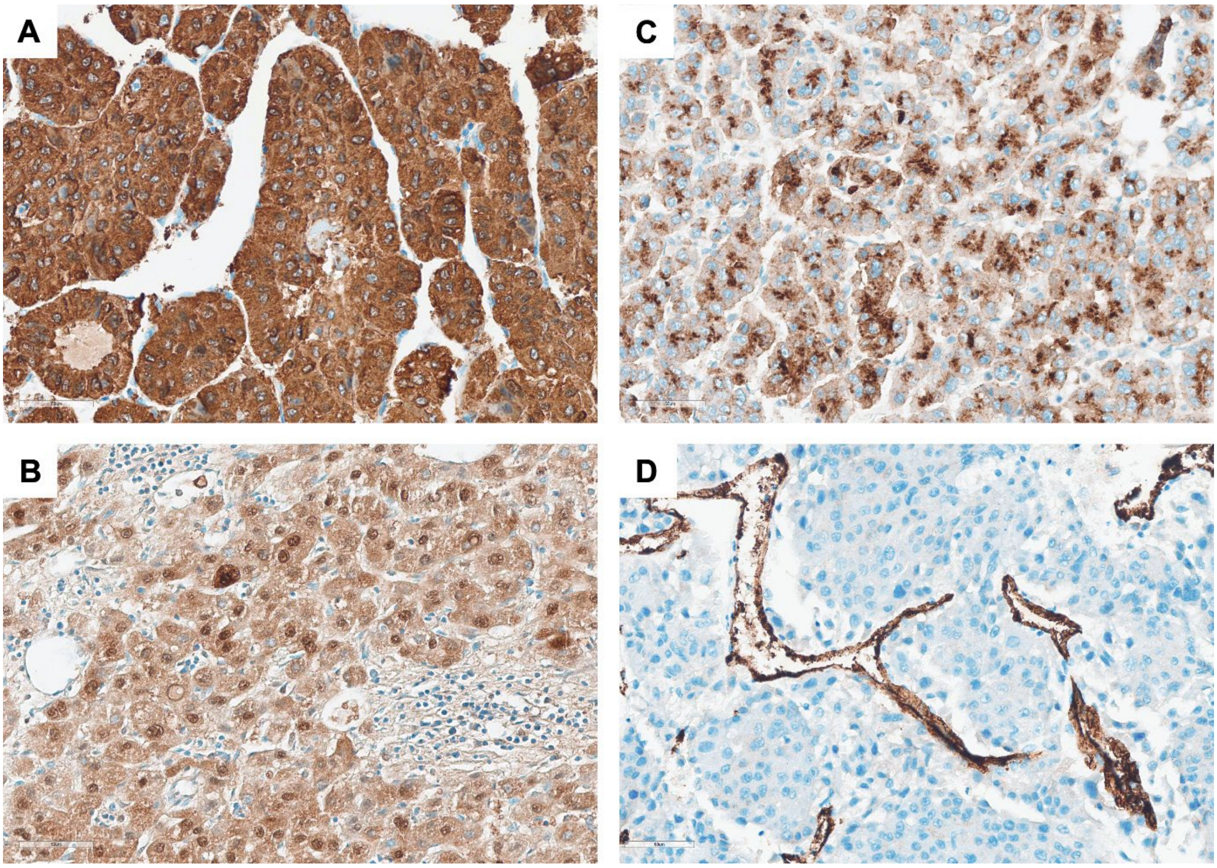

A case was considered positive if at least one core showed positive staining

Fig. S2. Venn Diagram of PSMA, Glypican 3, HSP70 and GS immunostainings in HCC cases from the test cohort (n=213).

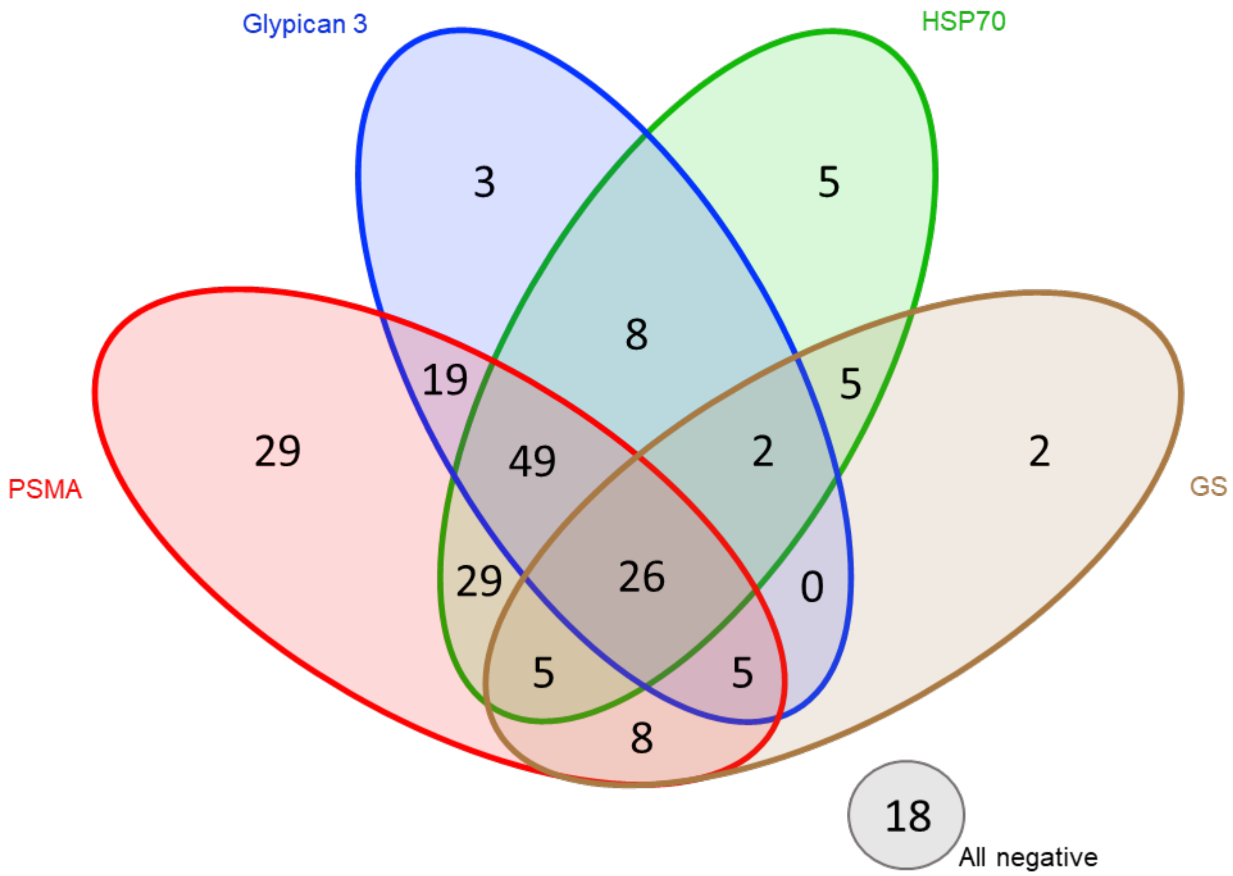

Fig. S3. PSMA expression in whole slides of paired surgical specimens (HCC and non-tumour liver) and biopsies from 15 cases.

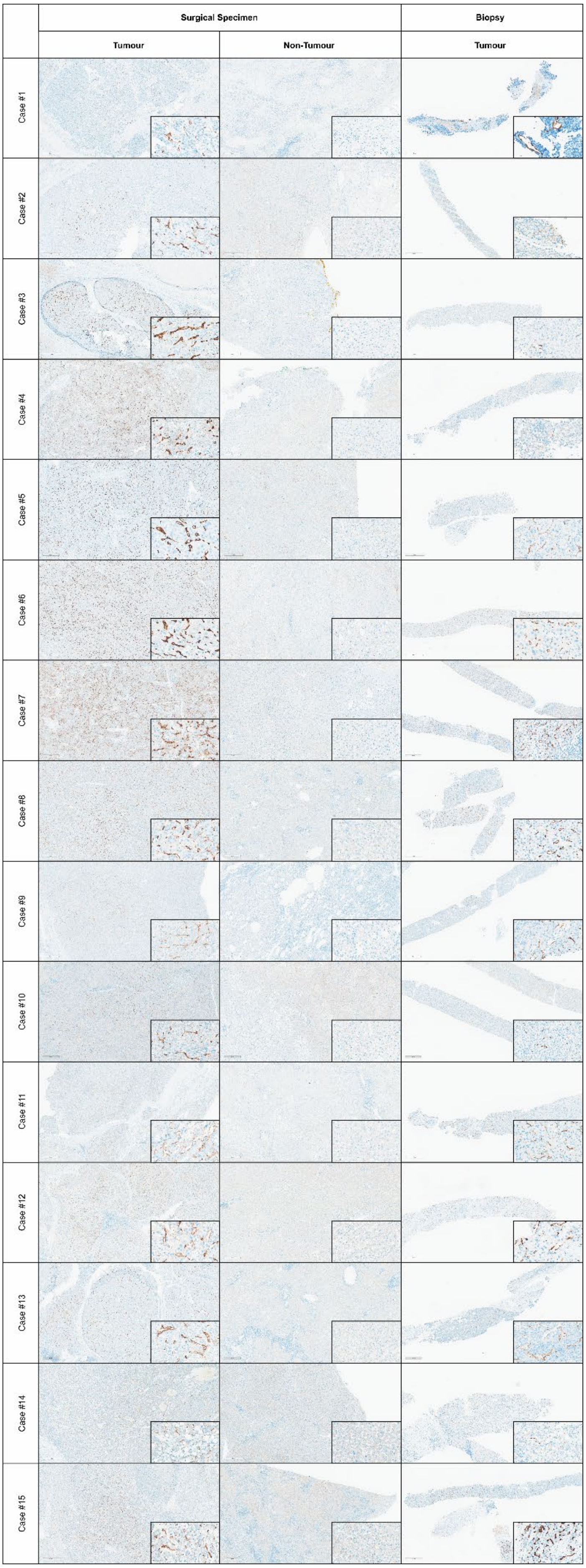
Surgical Specimen
Biopsy
Tumour
Non-Tumour
Tumour
Case #1
Case #2
Case #3
Case #4
Case #5
Case #6
Case #7
Case #8
Case #9
Case #10
Case #11
Case #12
Case #13
Case #14
Case #15
